# The DNA Repair Enzyme XPD Is Partially Regulated by PI3K/AKT Signaling in the Context of Bupivacaine-Mediated Neuronal DNA Damage

**DOI:** 10.1155/2021/9925647

**Published:** 2021-10-07

**Authors:** Wei Zhao, Lei Zeng, Jiaming Luo, Ji Li, Luying Lai, Shiyuan Xu, Zhongjie Liu

**Affiliations:** ^1^Department of Anesthesiology, Zhujiang Hospital, Southern Medical University, 253 Industrial Road, Guangzhou City, Guangdong Province, China; ^2^Department of Laboratory Medicine, Zhujiang Hospital, Southern Medical University, 253 Industrial Road, Guangzhou City, Guangdong Province, China

## Abstract

Bupivacaine, a local anesthetic widely used for regional anesthesia and pain management, has been reported to induce neuronal injury, especially DNA damage. Neurons employ different pathways to repair DNA damage. However, the mechanism underlying bupivacaine-mediated DNA damage repair is unclear. A rat neuronal injury model was established by intrathecal injection of (3%) bupivacaine. An in vitro neuronal injury model was generated by exposing SH-SY5Y cells to bupivacaine (1.5 mmol/L). Then, a cDNA plate array was used to identify the DNA repair genes after bupivacaine exposure. The results showed that xeroderma pigmentosum complementary group D (XPD) of the nuclear excision repair (NER) pathway was closely associated with the repair of DNA damage induced by bupivacaine. Subsequently, Western blot assay and immunohistochemistry indicated that the expression of the repair enzyme XPD was upregulated after DNA damage. Downregulation of XPD expression by a lentivirus aggravated the DNA damage induced by bupivacaine. In addition, phosphatidyl-3-kinase (PI3K)/AKT signaling in neurons was inhibited after exposure to bupivacaine. After PI3K/AKT signaling was inhibited, bupivacaine-mediated DNA damage was further aggravated, and the expression of XPD was further upregulated. However, knockdown of XPD aggravated bupivacaine-mediated neuronal injury but did not affect PI3K/AKT signaling. In conclusion, the repair enzyme XPD, which was partially regulated by PI3K/AKT signaling, responded to bupivacaine-mediated neuronal DNA damage. These results can be used as a reference for the treatment of bupivacaine-induced neurotoxicity.

## 1. Introduction

Local anesthesia (LA) has been widely used for surgical anesthesia and short- and long-term pain management since 1884 [1]. An amide-type local anesthetic, bupivacaine, is one of the most widely used local anesthetics for during labor and postoperative pain management. Previous studies have shown that bupivacaine might be neurotoxic, even at clinically relevant concentrations [2, 3]. However, the exact mechanism underlying the neurotoxicity of bupivacaine is unclear.

DNA breakage arising from oxidative damage is a significant threat to the genome stability in mature neurons [4, 5]. Our previous studies also revealed that oxidative stress is essential for neurotoxicity induced by bupivacaine [6, 7]. Oxidative stress is a crucial factor in DNA damage. Repair of those DNA lesions caused by oxidative stress requires the activity and interaction of different DNA repair pathways [8, 9], including base excision repair (BER), nuclear excision repair (NER), strand break (single- and double-stranded) repair, and homologous recombination (HR). DNA damage repair mechanisms are known to play an essential role in bupivacaine-mediated neurotoxicity. However, it is not clear which repair pathways are primarily involved in the pathological process of bupivacaine neurotoxicity. Therefore, studying the exact repair mechanism is necessary to prevent neuronal DNA damage mediated by bupivacaine.

Herein, a cDNA microplate array was used after SH-SY5Y cells were exposed to bupivacaine. The data revealed that the expression of XPD(/ERCC2), the gene that is essential for the NER pathway, is significantly increased. Subsequently, in vivo and in vitro experiments verified that the DNA repair enzyme XPD plays an essential role in bupivacaine-mediated neurotoxicity. The critical enzyme XPD may be closely involved in the repair of bupivacaine-mediated neuronal DNA damage. However, how XPD is regulated in the context of bupivacaine-mediated neuronal DNA damage remains unknown. The phosphatidyl-3-kinase (PI3K)/AKT signaling pathway is essential for protecting neuronal cells from oxidative stress [10]. According to our previous proteomics study, PI3K is inhibited in the context of bupivacaine-mediated neurotoxicity [7]. Although PI3K signaling has been reported to play an essential role in regulating bupivacaine-mediated neurotoxicity, the association between XPD and PI3K signaling is unknown.

Therefore, the present study had two specific aims: (1) to explore whether and verify that XPD is the key for bupivacaine-mediated neuronal DNA damage repair and (2) to determine the possible mechanism by which XPD is regulated by the PI3K signaling pathway.

## 2. Materials and Methods

### 2.1. Chemicals and Reagents

The bupivacaine hydrochloride was purchased from Sigma Chemical Co. (St. Louis, MO). Dulbecco's modified Eagle's medium (DMEM)/F12, fetal bovine serum, and penicillin and streptomycin were purchased from Thermo Fisher Scientific (Waltham, MA, USA). The pancreatic enzyme (including or excluding) EDTA was purchased from Gibco, USA. LY294002 was purchased from SelleckChem, USA.

### 2.2. Cell Culture and Treatment

The SH-SY5Y cell line was purchased from the Chinese Academy of Sciences (Shanghai, China). SH-SY5Y cells were held at 37°C in 5% CO_2_ in DMEM/F12 medium supplemented with 10% FBS and 1% penicillin/streptomycin. The culture medium was replaced daily during cell growth. Cells were grown in a 75 mm flask and subcultured in 6-well (seeding density 5.0 × 10^5^ cells) or 12-well (planting density of 1.0 × 10^5^ cells) plates. Experiments were conducted when cells reached 85% confluence. SH-SY5Y cells were treated with LY294002 (10 *μ*M) for 2 h before bupivacaine stimulation [11]. Following the termination of cell culture, the cells and medium were collected and stored at -80°C until analysis.

### 2.3. Animals and Treatment

Age-matched 10-week-old male Sprague-Dawley rats weighing approximately 250 g at the commencement of the experiment were supplied by the center of animals of Southern Medical University, Guangzhou. Rats were kept in a temperature-controlled environment with access to food and water. All animal procedures were approved by and performed in compliance with the ethical guidelines of the Animal Ethics Committee of Southern Medical University.

Intrathecal catheterization was performed as previously described [12]. The L4~5 intervertebral space was selected to insert the PE10 catheter (Smith Medical, UK). The rats from the model group were intrathecally administered 3% bupivacaine 20 *μ*L. In contrast, rats in the control groups were administered 20 *μ*L of normal saline. All injections were performed on a Wednesday or Friday between 2 and 4 pm to avoid possible time-dependent variations in pharmacokinetics. Before the test, rats were allowed at least two days to rest for recovery from the operation. Rats having any problem with tail movements or motor dysfunction in the hind limbs were not used in the ensuing experiments.

### 2.4. Mechanical/Thermal Thresholds

All animals were tested for mechanical thresholds and thermal thresholds as described [13] before and after injection of 3% bupivacaine. All behavioral testers were blinded to the experimental grouping information. Mechanical thresholds were assessed by measuring the paw withdrawal threshold (PWT) with a set of Von Frey filaments (0.04-0.2 g; Ugo Basile, Gemonio, Italy). The filament was applied to the plantar surface of the left hind paw at a vertical angle for up to 3 s from the bottom. Fifty percent of mechanical withdrawal threshold (MWT) values were determined using the up-down method.

Thermal thresholds were assessed by measuring the paw withdrawal latency to radiant heat stimuli. Each animal was placed in elevated chambers on a Plexiglas floor and acclimated to the testing environment for 30 minutes before the experiments. The radiant heat source (plantar test, 37370; Ugo Basile, SRL, Gemonio, Italy) was applied to the center of the plantar surface of the left hind paw with at least a 3-minute interval. The average withdrawal latency of the trials was recorded as the response latency.

### 2.5. TUNEL Assay

After 24 h treatment with bupivacaine, the frozen transverse sections from the lumbar enlargement of the spinal cord from every group were acquired. According to manufacturer's instructions, sections were tested with In Situ Cell Death Detection Kit, Fluorescein (Roche Diagnostics GmbH, Germany). Immunocytochemistry analysis of mouse primary neuron cells labeling NeuN with ab177487 (Abcam, Cambridge, MA, USA) is at 1/100. Goat Anti-Rabbit IgG H&L (Alexa Fluor® 594) (ab150080) at 1/1000 was used as the secondary antibody (Red). The fluorescence images were visualized under a fluorescence microscope (Carl Zeiss, Jena, Germany). The TUNEL-positive neurons were identified by colocalizing the TUNEL signal (green fluorescence) and NeuN (red fluorescence). The total number of TUNEL positive neurons in the spinal cord was counted from each slide.

### 2.6. Cell Viability

SH-SY5Y cells were seeded at a density of 1 × 10^4^ cells per well in a 96-well cell culture plate, and cell viability was determined with the Cell Counting Kit-8 (CCK8, Dojindo, Japan) according to manufacturer's instructions as previously described [14]. Briefly, after exposure to bupivacaine (0, 0.5, 1.0, 1.5, 2.0, and 2.5 mM) for 24 h, CCK-8 solution (10 *μ*L/well) was added to the SH-SY5Y cell culture medium. Then, the 96 well-plate was incubated for an additional 4 h at 37°C, according to the manufacturer. The optical density of the homogeneous purple solutions was measured at 450 nm using a spectrophotometer. The cell half-maximal inhibitory concentration (IC50) was calculated using the GraphPad software [15].

### 2.7. Detection of Lactate Dehydrogenase (LDH)

The levels of LDH in the cell culture medium released from injured SH-SY5Y cells were determined by the LDH Cytotoxicity Assay kit (Invitrogen, USA) following manufacturer's instruction. Briefly, the SH-SY5Y cells were plated in 6-well plates for 24 h. After being treated with bupivacaine (0, 0.5, 1.0, 1.5, 2.0, and 2.5 mM), the cell culture medium was collected from SH-SY5Y cells and assayed for LDH levels. The absorbance at a wavelength of 450 nm was measured and quantified.

### 2.8. cDNA Plate Array

Signosis's proprietary cDNA plate array is a plate-based hybridization profiling analysis that monitors the expression of dozens of genes through reverse transcription of mRNA into cDNA [16]. About 10^4^-10^5^ SH-SY5Y cells with (as an experimental group) and without (as control group) exposure to 1.5 mM bupivacaine were washed with 200 *μ*L ice-cold 1× PBS and add 100 *μ*L ice-cold cell lysis buffer and then subject to snap-frozen at -80°C. The 20 *μ*L cDNA was synthesized from each sample and labeled with biotin and ready for hybridization on the plate. Targeted genes are then specifically captured onto individual wells on a scale, instead of membranes, through a precoated gene-specific oligonucleotide. Then, the captured cDNAs are tested with streptavidin-HRP. The microplate luminometer was used to report the relative light units (RLUs). The expression level of genes is directly proportional to the luminescent intensity.

### 2.9. Heatmap and Clustering

The heatmap, a graphical representation of data with the individual values in a matrix, was represented as grids of colors plus clustering on both rows and columns. The heatmap-2 function in R based on the cDNA Plate Assay data was used to generate heatmaps of differentially expressed genes [17]. Samples were divided into different clusters using Ward's hierarchical clustering method, and the expression of genes was shown in colors in the center of the heatmap. The heatmap was available from the package “gplot” as an enhanced version or its basic function stats in R ted. (https://CRAN.R-project.org/package=gplots).

### 2.10. Western Blotting

Tissue or cells were lysed in a lysis buffer by sonication. After centrifugation, the protein was collected. The protein concentration was determined by the Bradford method and proceeded for Western blotting. An equal amount of protein extracts was separated by 10% SDS-PAGE and transferred to PVDF membranes (Immobilon-P, Millipore, Bedford, MA, USA). The blots were blocked with 5% BSA at room temperature for 1 hour and incubated overnight at 4°C with primary antibodies against p-*γ*-H2AX (rabbit, 1 : 1000; cell signaling), Bax and Bcl-2 (rabbit, 1 : 1000; cell signaling), XPD (rabbit, 1 : 1000; cell signaling), PI3K, AKT, p-AKT (rabbit, 1 : 1000; cell signaling), p-PI3K (rabbit,1 : 1000, Abcam), and *β*-actin (rabbit, 1 : 2000; cell signaling). Then, these blots were incubated with HRP-conjugated secondary antibody, developed in ECL solution, and exposed onto hyperfilm (Amersham Biosciences) for 1-10 min. Specific bands were evaluated by apparent molecular size. The protein abundance was most often measured using an optical density (OD) algorithm. The intensity of the selected bands was captured and quantified by a densitometric method used by the Image J software 1.48a (http://imagej.nih.gov).

### 2.11. Immunohistochemistry

After treatment with bupivacaine for 24 h, the lumbar spinal enlargement of rats was obtained.

Immunohistochemistry was performed to detect the expression of XPD in the spinal cord tissue. Briefly, samples were fixed with 4% polyoxymethylene, embedded in paraffin, and sectioned at a thickness of 5 *μ*m. Then, the sections were dewaxed in xylene and rehydrated in graded ethanol, followed by incubation in H_2_O_2_ (3%) for 10 min to block endogenous peroxidase. Then, sections were incubated with anti-XPD at 4°C overnight, followed by incubation with the appropriate secondary antibodies for 45 min at room temperature. The sections were stained with hematoxylin and observed under a microscope (Olympus-IX51; Olympus Corporation, San Jose, CA, USA). Integrated optical density (IOD) of the positively stained area was measured immunohistochemical of spinal cord tissue. The XPD expression level in the spinal cord was represented by integral optic density (IOD). The selected images were then converted into an 8-bit grayscale. Then, the IODs of each image were counted and measured using Image-Pro Plus v6.0 software (Media Cybernetics Inc., Bethesda, MD, USA).

### 2.12. Flow Cytometry

Flow cytometric analyses were carried out to detect the percentages of apoptotic cells. After exposure to bupivacaine for 24 h, a total of 5 × 10^5^ SH-SY5Y cells were collected and assayed by the FITC Annexin V Apoptosis Detection Kit (BD Pharmingen) following manufacturer's instruction. Briefly, cells were incubated with 5 *μ*L Annexin V-FITC for 20 min. After removing the media, cells were incubated with 5 *μ*L propidium iodide (PI). Gently vortex the cells and incubate for 15 min at RT (25°C) in the dark. Cells samples were analyzed by the flow cytometer (BD Biosciences FACS Calibur, USA) within 1 h.

### 2.13. Comet Assay

The comet assay was used to detect DNA damage in individual cells as previously described [18]. Briefly, the SH-SY5Y cells were plated in 12-well plates, and the extent of DNA damage was measured by the kit (Trevigen's Comet Assay® Kit) following manufacturer's instructions. Images were acquired on a Carl Zeiss fluorescence microscopy (Jena, Germany). At least 50 randomly selected cells were analyzed by the Comet Assay Software Project.

### 2.14. Lentivirus-Mediated Gene Silencing of XPD

According to the cDNA sequence of XPD (Target Seq: TGGCCCTGATCATGGCATA), shRNA was designed and synthesized, which was then annealed into the hU6-MCS-CMV-EGFP vector. After being identified by sequencing, hU6-MCS-CMV-EGFP vector and packaging vector were cotransfected into SH-SY5Y cells. Seventy-two hours later, the recombinant lentivirus-mediated gene silencing of XPD was obtained after harvesting and concentrating as previously described [18]. The fluorescence intensity was obtained 48 h after the cells were transfected with lentivirus. These images were used to determine the efficiency of lentivirus infection (shown as Supplemental Figure 1 A). Then, Q-PCR was used to detect mRNA levels of XPD after lentivirus infection in SH-SY5Y cells (shown as Supplemental Figure 1 B).

### 2.15. Statistical Analysis

All the data were represented as mean ± SD. The graphs and statistical data were completed by GraphPad Prism (GraphPad Software 8.0, Inc., La Jolla, CA). The statistical significance was determined by one-way analysis of variance (ANOVA) followed by the Tukey-Kramer test. The differences of *p* < 0.05 were considered statistically significant.

## 3. Results

### 3.1. Bupivacaine-Induced Apoptosis in Rat Spinal Cord Tissue and Behavioral Changes In Vivo

**Figure 1:**
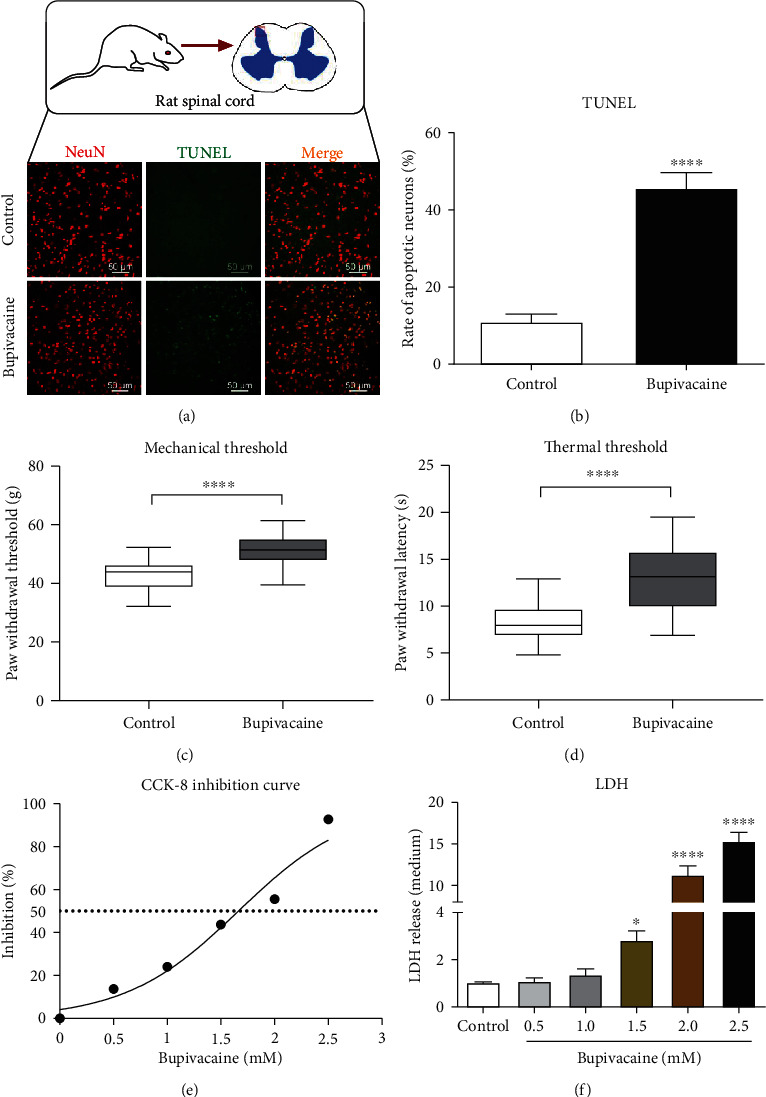
Bupivacaine causes neurotoxic damage. In vivo, bupivacaine-induced apoptotic damage in rat spinal cord tissue and behavioral changes. The rats in the neuronal damage model group were intrathecally administered 20 *μ*L of 3% bupivacaine, while rats in the control group were administered normal saline. Twenty-four hours after treatment with bupivacaine, the TUNEL staining was performed to determine the cell apoptosis rate ((a, b) *n* = 6; ^∗∗∗∗^*p* = 0.0001) in spinal cord tissue slices. NeuN was used to label mature neurons. TUNEL/NeuN positive cells (a) represent apoptotic neurons. The PWMT and PWTL were increased in the bupivacaine-treated group compared with the control group ((c, d) *n* = 6; ^∗∗∗∗^*p* = 0.0001). The in vivo data are shown as the mean ± SD. Bupivacaine may induce SH-SY5Y cell neurotoxicity in vitro. After SH-SY5Y cells were exposed to different concentrations of bupivacaine, the dose inhibition curve was plotted ((e) *n* = 3), and the IC50 of bupivacaine was calculated as 1.5 mmol/L. The release of LDH was determined to assess cytotoxicity ((f) *n* = 3; ^∗^*p* = 0.0394 and ^∗∗∗∗^*p* < 0.0001 vs. the control group). The data are shown as the mean ± SD.

Accumulating evidence has shown that the local anesthetics (bupivacaine) are potentially neurotoxic and that neurologic impairment after spinal anesthesia may result from the direct neurotoxic effect of bupivacaine [19]. As shown in Figure 1, TUNEL staining revealed that after intrathecal application of bupivacaine (3%), the percentage of apoptotic neurons (Figures 1(a) and 1(b); *n* = 6; ^∗∗∗∗^*p* = 0.0001) in spinal cord tissue slices was significantly increased. The data showed that bupivacaine induced neuronal apoptosis in rat spinal cord tissue. Furthermore, behavioral tests for mechanical and thermal thresholds were assessed before and 24 h after bupivacaine injection. The paw withdrawal mechanical threshold (PWMT) (Figure 1(c); ^∗∗∗∗^*p* = 0.0001) and paw withdrawal threshold latency (PWTL) (Figure 1(d); ^∗∗∗∗^*p* = 0.0001) were significantly increased in the bupivacaine group compared with the control group. These data showed that bupivacaine induced behavioral changes in rats.

### 3.2. Bupivacaine-Induced Neurotoxic Injury in SH-SY5Y Cells

SH-SY5Y cells are undifferentiated human neuroblastoma cells [20]. After exposure to different concentrations (0.5, 1.0, 1.5, 2.0, and 2.5 mM) of bupivacaine, the CCK-8 assay was used to assess SH-SY5Y cell viability. Then, the dose inhibition curve was plotted (Figure 1(e)). The half inhibitory concentration (IC50) of bupivacaine was approximately 1.5 mM. LDH is a marker of cytotoxicity. Compared with control treatment, bupivacaine induced SH-SY5Y cell cytotoxicity in a dose-dependent manner. Bupivacaine at 1.5 mM concentration started to demonstrate significant cytotoxicity compared to the control group (Figure 1(f); *n* = 3; ^∗^*p* = 0.0394, ^∗∗∗∗^*p* < 0.0001). Thus, 1.5 mM was selected as the concentration of bupivacaine for further in vitro studies.

### 3.3. Identification of DNA Repair Pathways In Vitro by a cDNA Microplate Array

**Figure 2:**
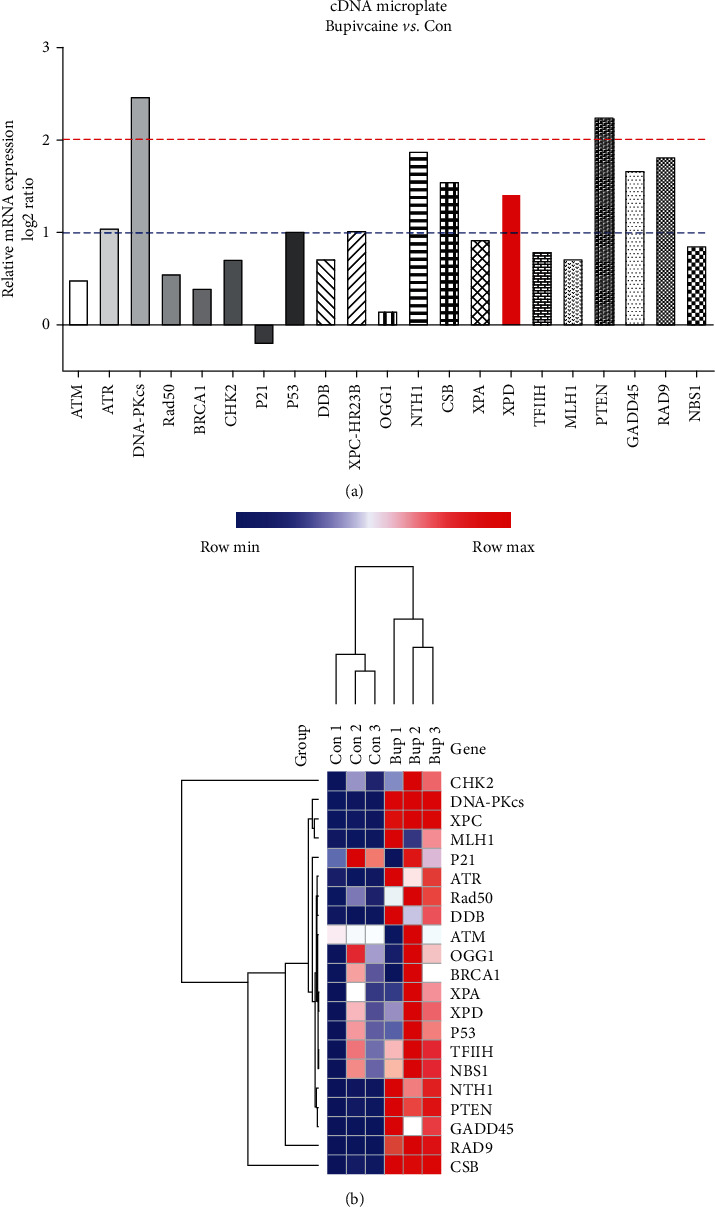
The expression of DNA damage repair genes was determined with a cDNA plate array after SH-SY5Y cells were exposed to bupivacaine. As shown in (a), a ratio > 0 indicates that the expression of the indicated gene was upregulated in the bupivacaine (Bup) group compared with the control (C) group, and a ratio < 0 indicates that the expression of the gene was downregulated. A ratio greater than 1 or less than -1 indicated that there was a one order of magnitude difference. A ratio greater than 2 or less than -2 indicated that there were two orders of magnitude difference. A heatmap of the 21 DNA repair proteins is shown in (b). Each column represents a sample (control group: Con1~3; bupivacaine group: Bup1~3), and each row represents one of the DNA repair proteins. The cDNA levels of the DNA repair genes are indicated by the different colors, with the color changing from green to red with increasing expression.

Accumulating evidence suggests that DNA damage is repaired by overlapping multiple DNA repair pathways [21]. Repair of these DNA lesions induced by bupivacaine may also require the activity and interaction of different DNA repair pathways, including BER and NER [22, 23]. To investigate the critical enzymes associated with DNA damage repair pathways, a cDNA plate array was used to measure DNA repair gene expression in SH-SY5Y cells treated with bupivacaine. Cells were exposed to 1.5 mM bupivacaine, and a cDNA plate array was used to detect the mRNA expression of DNA repair genes. As shown in Figure 2(a), a ratio > 0 indicated that the expression of the indicated gene was upregulated in the bupivacaine group compared with the control group, while a ratio < 0 indicated that the expression of the gene was downregulated. A ratio greater than 1 or less than -1 indicated that there was a one order of magnitude difference. A ratio greater than 2 or less than -2 meant there were two orders of magnitude differences. The cDNA microplate array results showed that bupivacaine resulted in differential expression of the following genes (Table 1): DNA-PKcs, PTEN, NTH1, RAD9, CSB, GADD45, XPD, XPC-HR23B, and P53.

Bidirectional hierarchical clustering heatmaps were made based on the identified DNA repair genes' cDNA levels. As shown in Figure 2(b), the samples could be separated by group (control and bupivacaine) according to cDNA expression, indicating the changes in the expression of the identified genes related to treatment whit bupivacaine. Furthermore, the identified repair genes were mainly associated whit the NER pathways.

### 3.4. The Expression of the NER Pathway-Related Gene XPD Was Significantly Increased after Bupivacaine-Induced DNA Damage In Vivo and In Vitro

**Figure 3:**
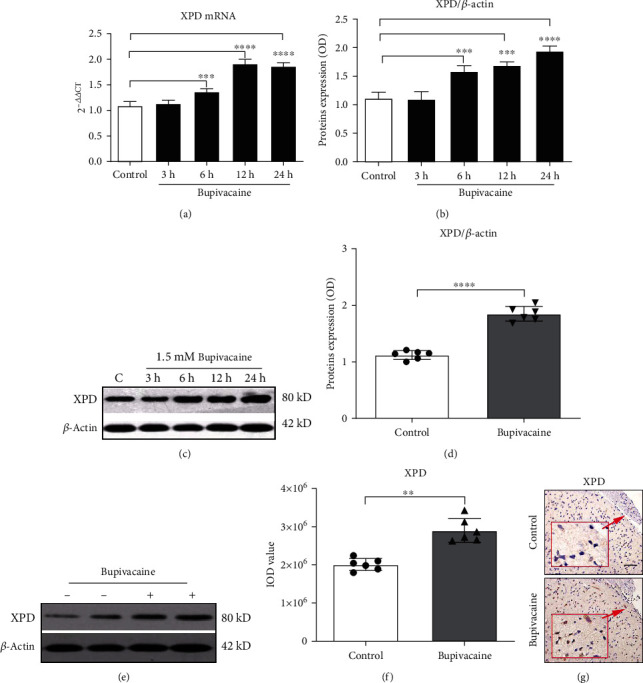
The expression of the crucial NER pathway-associated repair enzyme XPD was significantly increased after exposure to bupivacaine in vivo and in vitro. The mRNA expression of XPD (a) was significantly increased in bupivacaine-treated cells compared with control cells at different time points, especially at 12 h ((a) *n* = 3; ^∗∗∗^*p* = 0.0035, ^∗∗∗∗^*p* < 0.0001), and the protein expression of XPD was also significantly increased at different time points, especially at 24 h ((b, c) *n* = 3; ^∗∗∗^*p* = 0.001 and ^∗∗∗∗^*p* < 0.0001 vs. the control group). The expression of XPD in spinal cord tissue was significantly increased in the rat spinal neurotoxicity model compared with the control group (Western blotting: (d, e) *n* = 6; ^∗∗∗∗^*p* < 0.0001; immunohistochemistry: (f, g) *n* = 6; ^∗∗^*p* = 0.0028). The in vivo data are presented as the mean ± SD.

Q-PCR was used to test the mRNA expression of XPD over time after exposure to 1.5 mM bupivacaine in vitro. Additionally, Western blotting was used to measure the protein expression of XPD. Compared with control treatment, bupivacaine treatment significantly increased the mRNA expression of XPD (Figure 3(a); ^∗∗∗^*p* = 0.0035, ^∗∗∗∗^*p* < 0.0001) at different time points, especially at 12 h. The protein expression of XPD was also significantly increased at different time points, especially at 24 h (Figures 3(b) and 3(c); ^∗∗∗^*p* = 0.001, ^∗∗∗∗^*p* < 0.0001 vs. the control group).

Western blotting was used to evaluate the relative expression of XPD in vivo in rat spinal cord tissue (Figures 3(d) and 3(e); ^∗∗∗∗^*p* < 0.0001), while immunohistochemical was used to assess the relative expression of XPD in rat spinal cord tissue (Figures 3(f) and 3(g); ^∗∗^*p* = 0.0028). The data showed that the expression of XPD was significantly increased in the bupivacaine-treated group compared with the control group.

### 3.5. Knockdown of XPD Expression Significantly Aggravated Bupivacaine-Induced Neuronal DNA Damage and Neurotoxicity

**Figure 4:**
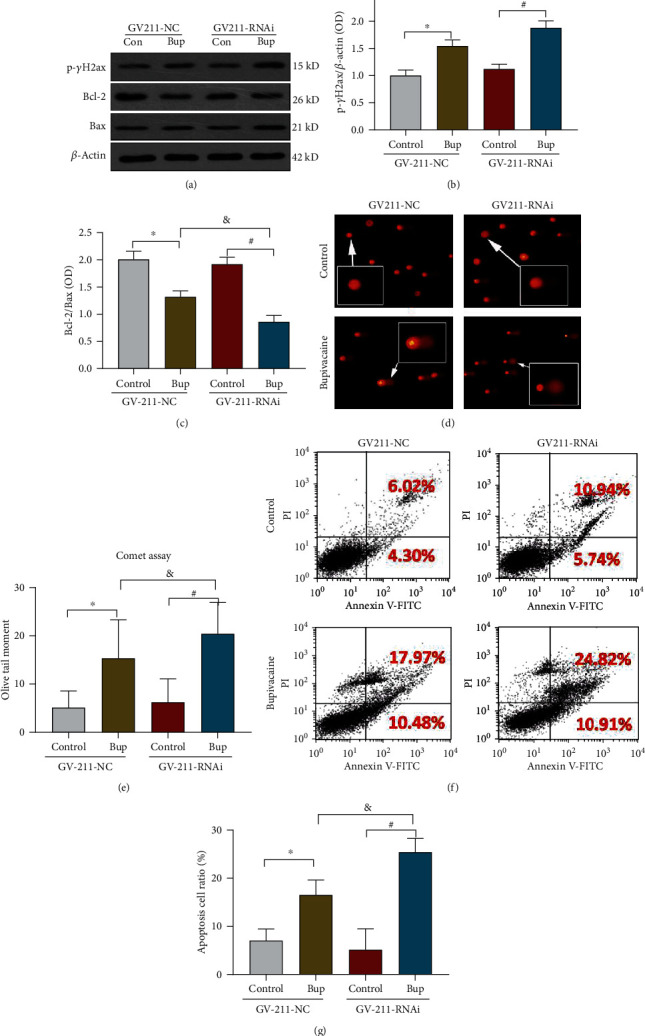
Downregulation of XPD expression further aggravated the neurotoxicity caused by bupivacaine. After XPD expression was downregulated with the XPD-GV211-RNAi-expressing lentivirus, the SH-SY5Y cell apoptosis and DNA damage induced by bupivacaine were exacerbated. GV211-NC served as a control lentivirus. DNA damage was aggravated in bupivacaine-treated SH-SY5Y cells in which XPD expression was inhibited (Bup-GV211-RNAi) compared to the control group (Bup-GV211-NC), as the phosphorylation of *γ*-H2AX, a DNA damage marker, was significantly increased ((a, b) *n* = 3; ^&^*p* = 0.0243), (^∗^*p* = 0.0013, and ^#^*p* = 0.0243, vs. the control group). Furthermore, the expression of the apoptosis-related proteins Bcl-2 and Bax was reduced ((a, c) *n* = 3; ^&^*p* = 0.0100) (^∗^*p* = 0.0008, and ^#^*p* < 0.0001 vs. the control group), and the olive tail moment was significantly higher in the comet assay ((d, e) *n* = 3; ^&^*p* = 0.0002) (^∗^*p* < 0.0001, and ^#^*p* < 0.0001 vs. the control group). Moreover, suppression of XPD expression significantly increased the apoptosis ratio, as determined by flow cytometry ((f, g) *n* = 3; ^&^*p* = 0.0308) (^∗^*p* < 0.0412, and ^#^*p* < 0.0003 vs. the control group). The data are shown as the mean ± SD of three independent experiments.

Cytotoxicity and DNA damage induced by bupivacaine were aggravated after XPD knockdown. Bupivacaine-treated SH-SY5Y cells were infected with an XPD-GV211-RNAi-expressing lentivirus. GV211-NC was used as a control lentivirus. DNA damage was aggravated in bupivacaine and GV211-RNAi-treated SH-SY5Y cells compared with bupivacaine and GV211-NC-treated SH-SY5Y cells. The data showed that in the comet assay, the olive tail moment was significantly higher (Figures 4(d) and 4(e); ^&^*p* = 0.0002); (^∗^*p* < 0.0001, and ^#^*p* < 0.0001 vs. the control group), while phosphorylation level of *γ*-H2AX, a marker of DNA damage, was significantly increased (Figures 4(a) and 4(b); ^&^*p* = 0.0243); (^∗^*p* = 0.0013, and ^#^*p* = 0.0243 vs. the control group). In addition, the ratio of the apoptosis-related protein Bcl-2/Bax was reduced (Figures 4(a) and 4(c); ^&^*p* = 0.0100); (^∗^*p* = 0.0008, and ^#^*p* < 0.0001 vs. the control group), and apoptosis was significantly increased as determined by flow cytometry (Figures 4(f) and 4(g); ^&^*p* = 0.0308); (^∗^*p* < 0.0412, and ^#^*p* < 0.0003 vs. the control group).

### 3.6. Inhibition of PI3K/AKT Signaling Aggravated Bupivacaine-Induced Neuronal DNA Damage and Upregulated the Expression of XPD

Previous studies have shown that the PI3K/AKT pathway plays a vital role in oxidative neurotoxicity [24]. Our latest data also showed that PI3K/AKT is closely involved in bupivacaine-induced neurotoxicity [7]. Furthermore, XPD can inhibit tumor cell growth and invasion by regulating the PI3K/AKT pathway [25]. The relationship between the XPD and PI3K/AKT pathways in the bupivacaine-mediated oxidative neurotoxicity is unclear.

**Figure 5:**
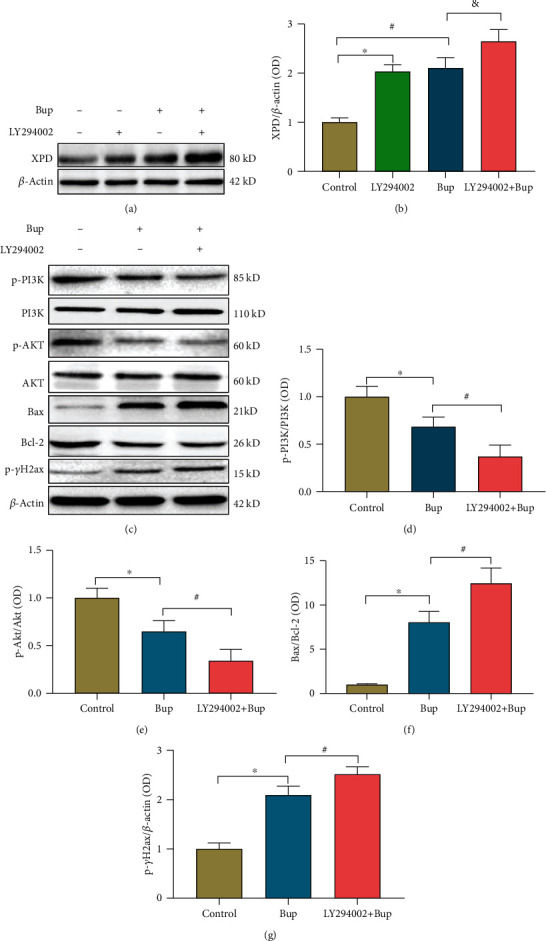
Inhibition of PI3K/AKT aggravated SH-SY5Y injury caused by bupivacaine and further increase the expression of XPD. Treatment with LY294002, a PI3K/AKT inhibitor, alone significantly increased XPD expression in SH-SY5Y cells ((a, b) *n* = 3; ^∗^*p* = 0.0005). Moreover, the expression of XPD was further increased in the bupivacaine and LY294002-treated group compared with the bupivacaine-treated group ((a, b) *n* = 3; ^&^*p* = 0.0254) (^#^*p* = 0.0003 vs. the control group). LY294002 (10 *μ*M) further inhibited the protein expression of p-PI3K ((c, d) *n* = 3; ^#^*p* = 0.0316) (^∗^*p* = 0.0308 vs. the control group) and p-AKT ((c, e) *n* = 3; ^#^*p* = 0.0346) (^∗^*p* = 0.0198 vs. the control group) after bupivacaine treatment. The expression of the apoptosis-related proteins Bax and Bcl-2 and p-*γ*H2AX (markers of DNA damage) was increased in the 10 *μ*M LY294002 and bupivacaine-treated group compared with the bupivacaine group ((f) ^#^*p* = 0.0115; (g) *n* = 3; ^#^*p* = 0.0360) ((f) ^∗^*p* = 0.0011 vs. the control group) ((g) ^∗^*p* = 0.0003 vs. the control group). The data are shown as the mean ± SD of three independent experiments.

PI3K/AKT signaling was inhibited, and bupivacaine was administered to induce neuronal DNA damage. We utilized the inhibitor LY294002 (10 *μ*M) to suppress the PI3K/AKT pathway [26]. LY294002 treatment alone significantly increased XPD expression in SH-SY5Y cells (Figures 5(a) and 5(b); ^∗^*p* = 0.0005). Moreover, the expression of XPD was further increased in the LY294002 and bupivacaine treated group compared with the control group (Figures 5(a) and 5(b); ^&^*p* = 0.0254). LY294002 was used to suppress the activation of the PI3K/AKT signaling pathway in many previous studies [27]. Here, LY294002 (10 *μ*M) further inhibited the protein expression of p-PI3K (Figures 5(c) and 5(d); *n* = 3; #*p* = 0.0316) and p-AKT (Figures 5(c) and 5(e); *n* = 3; ^#^*p* = 0.0346) after bupivacaine treatment. Furthermore, the expression of the apoptosis-related proteins Bax/Bcl-2 and the DNA damage marker p-*γ*H2AX was further increased in the LY294002 and bupivacaine-treated group compared with the bupivacaine-treated group (Figure 5(f), ^#^*p* = 0.0115; Figure 5(g), ^#^*p* = 0.0360).

### 3.7. Knockdown of XPD Did Not Affect the PI3K/AKT Pathway

**Figure 6:**
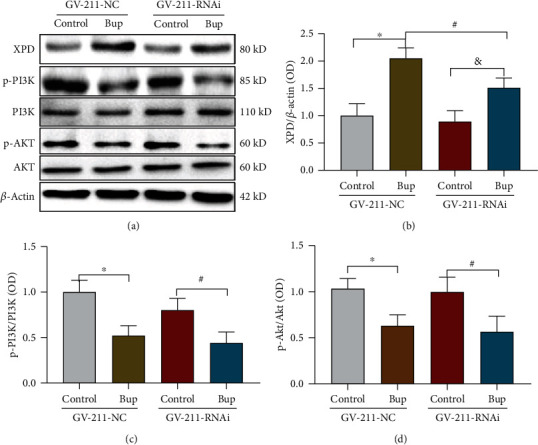
Downregulation of XPD expression did not affect the PI3K/AKT pathway. In SH-SY5Y cells, bupivacaine increased the expression of XPD ((a, b) *n* = 3; ^∗^*p* = 0.0009 and ^&^*p* = 0.0208 vs. the control group). The expression of XPD was downregulated by the XPD-GV211-RNAi-expressing lentivirus, and GV211-NC served as a control lentivirus ((a, b) *n* = 3; ^#^*p* = 0.0413). p-PI3K and p-AKT expression was inhibited by bupivacaine ((a, c) ^∗^*p* = 0.0060 and ^#^*p* = 0.0291; (a, d) ^∗^*p* = 0.0345 and ^#^*p* = 0.0242 vs. the control group). Nevertheless, the expression of p-PI3K ((a, c) *n* = 3; *p* = 0.8535) and p-AKT ((a, d) *n* = 3; *p* = 0.9396) was not further reduced in the group in which XPD expression was downregulated compared with the GV-211-NC-treated group. The data are shown as the mean ± SD of three independent experiments.

In SH-SY5Y cells, bupivacaine increased the expression of XPD (Figures 6(a) and 6(b)). The expression of XPD was downregulated by the XPD-GV211-RNAi-expressing lentivirus (GV211-NC served as a control lentivirus group; Figures 6(a) and 6(b); ^#^*p* = 0.0413). p-PI3K (Figure 6(c); ^∗^*p* = 0.0060, ^#^*p* = 0.0291) and p-AKT (Figure 6(d); ^∗^*p* = 0.0345, and ^#^*p* = 0.0242; vs. the control group) expression was inhibited by bupivacaine. Nevertheless, the downregulation of XPD expression induced by the XPD-GV211-RNAi-expressing lentivirus did not further reduce p-PI3K and p-AKT compared with that in the bupivacaine group (Figure 6(c); *p* = 0.8535; Figure 6(d); *p* = 9396).

## 4. Discussion

Previous studies have shown that bupivacaine can cause neuronal oxidative DNA damage [7, 14]. Repair of oxidative DNA damage is mainly accomplished by excision repair mechanisms such as BER [8] and NER [28]. However, it remains unclear whether specific repair pathways and essential repair proteins are involved in repairing bupivacaine-induced oxidative DNA damage.

cDNA plate arrays can detect differences in mRNA expression levels among different treatment groups with high sensitivity [16]. Hundreds of repair enzymes are involved in repairing DNA damage caused by bupivacaine through a large and complex network of repair mechanisms. However, due to the limitations of the cDNA plate array, we were unable to detect all the genes involved in DNA damage repair. Only the essential DNA repair genes reported in the relevant literature could be studied. This is a limitation of the current study.

A cDNA plate array was used to identify regulatory and repair enzymes necessary for DNA damage repair in the context of bupivacaine-induced SH-SY5Y cytotoxicity. The results showed that bupivacaine caused differential expression of the following genes (Table 1): DNA-PKcs, PTEN, NTH1, RAD9, CSB, GADD45, XPD, XPC-HR23B, and P53. Furthermore, in a previous study, bupivacaine was shown to cause oxidative damage to DNA in SH-SY5Y cells. The DNA damage initiated the NER pathway. NER is a universal and versatile process that involves two subpathways: global genome NER (GG-NER) and transcription-coupled NER (TC-NER). XPD is the common repair enzyme of GG-NER and TC-NER pathways [29]. The expression of the vital repair enzyme XPD was increased, and this enzyme participated in repairing oxidative DNA damage (Figure 2). Our previous study [18] also suggested that XPD participates in repairing oxidative DNA damage caused by bupivacaine in neurons. Therefore, XPD may be the most critical restriction enzyme in NER, the primary DNA repair pathway in mammalian cells [29, 30]. Nevertheless, the critical regulatory mechanism of the repair enzyme XPD in bupivacaine-induced DNA damage is unknown.

The PI3K/AKT pathway is an intracellular signal transduction pathway that promotes metabolism, proliferation, cell survival, growth, and angiogenesis in response to extracellular signals [31]. Several studies have suggested that activation of PI3K/AKT signaling can inhibit neural damage. Lei and Chen found that resveratrol attenuates brain damage in permanent focal cerebral ischemia via PI3K/AKT signaling pathway activation in rats [32]. Deng et al. suggested that berberine protects rotenone-treated SH-SY5Y cells by activating the PI3K/AKT signaling pathway [33]. A previous study showed that the PI3K/AKT signaling plays a crucial role in activating and regulating different pathways in the context of bupivacaine-induced neurotoxicity [7]. Our results (Figure 5) also showed that the activation of PI3K/AKT signaling was decreased in SH-SY5Y cells after exposure to bupivacaine. Whether there is a relationship between XPD and PI3K/AKT signaling is unclear. PI3K/AKT signaling is vital in maintaining genomic stability through involving DNA damage repair and cell cycle regulation [34, 35]. Emerging data suggest that the activation of PI3K/AKT signaling promotes cell survival partly by regulating the DNA damage response (DDR) [36]. LY294002 blocks the recruitment of translesion synthesis polymerases at sites of the DNA damage, thus hindering the progression of the DNA replication fork, leading to replication stress and cell death [37]. In this study, LY294002 increased the expression of the apoptosis-related proteins Bax, BCL-2, and p-*γ*H2AX (markers of DNA damage) (Figures 5(e)–5(g)). Aggravation of DNA damage led to more of the repair enzyme XPD in the DNA repair process until neuronal apoptosis was induced. PI3K/AKT inhibition by LY294002 further increased the bupivacaine-induced expression of the repair enzyme XPD (Figures 5(a) and 5(b)). In contrast, downregulation of the XPD gene had no significant effect on PI3K/AKT signaling activation. These data indicated that the repair enzyme-XPD might be regulated by the PI3K/AKT signaling in response to DNA damage and plays a crucial role in combatting bupivacaine-induced neurotoxicity. Because XPD is expressed at low levels, the protein expression of XPD in the GV-211-RNAi-treated group was not significantly reduced. However, XPD expression was significantly upregulated after bupivacaine treatment. Thus, the effect of lentivirus was more obvious in bupivacaine-treated cells (as shown in Figure 6(b)). To verify the lentivirus knockdown efficiency, we obtained the fluorescence images of SH-SY5Y cells after infection with the GV-211-RNAi-expressing lentivirus and performed Q-PCR; the Q-PCR data are shown in Supplemental Figure 1. Therefore, a complete knockout strategy must be considered in future studies.

Bupivacaine, which is widely used clinically for regional nerve blockade and pain management [38], can induce neurotoxicity. Ruppen et al. showed large variations in the bupivacaine concentration in the cerebrospinal fluid (CSF) of patients who received spinal anesthesia [39]. Experimental studies in animal models have shown that the concentration at which bupivacaine causes neurotoxicity damage varies greatly. One criticism of the study may be the dose and concentration of local anesthetic used. Although a lower concentration of bupivacaine (0.5%-0.75%) is generally accepted as safe, previous studies have shown that bupivacaine might be neurotoxic at clinically relevant concentrations [2, 3]. Cauda equina syndrome, a severe neurological complication, has been associated with spinal anesthesia achieved with 0.5%-0.75% bupivacaine [40]. Because local anesthetic solutions rarely induce neurologic injury, larger dosages are required to observe their neurotoxic effects. A previous study showed that rats given 2.13% bupivacaine incurred only minimal morphologic damage without functional impairment. To study the neurotoxicity of bupivacaine, high concentrations that cause neuronal injury are usually used. The possible mechanism of injury was studied. In this study, 3% was selected as the bupivacaine concentration in the preliminary experiment [41, 42]. The degree of neuronal apoptosis is thought to play a role in pain behavior. Garraway et al. showed that neuronal apoptosis occurs at the same time as the onset of mechanical allodynia. In addition, the immunohistochemical analysis revealed distinct morphological signs of apoptosis in neurons at 24 h after stimulation [43]. This is consistent with the results in Figure 1(a).

SH-SY5Y neuroblastoma cells are a type of the human nervous system with a low degree of differentiation. Both differentiated and undifferentiated SH-SY5Y cells have been widely used in mechanistic studies on the pathogenesis, prevention, and treatment of central nervous system diseases [44, 45]. This paper is aimed at establishing a model simulating bupivacaine-induced neuronal injury in adult patients with differentiated and matured neurons. Thus, differentiated SH-SY5Y cells were more suitable than undifferentiated SH-SY5Y cells in this study. However, it has been found that undifferentiated SH-SY5Y cells are more sensitive to drug toxicity [46]. To study the neuronal damage caused by bupivacaine, we selected undifferentiated SH-SY5Y cells as cell models. SH-SY5Y cells, whether differentiated or undifferentiated, are nervous system tumor-derived cells. Thus, they cannot fully simulate the toxicity in normal neurons, which is a limitation of this study. The CCK-8 assay was used to measure cell viability, and the IC50 (1.5 mmol/L) of bupivacaine was calculated. When bupivacaine is administered at a concentration of 1–1.5 mmol/L [45, 47], nerve cells can be detected [45, 48]. Therefore, a bupivacaine concentration of 1.5 mmol/L was selected as the drug concentration for further study.

**Figure 7:**
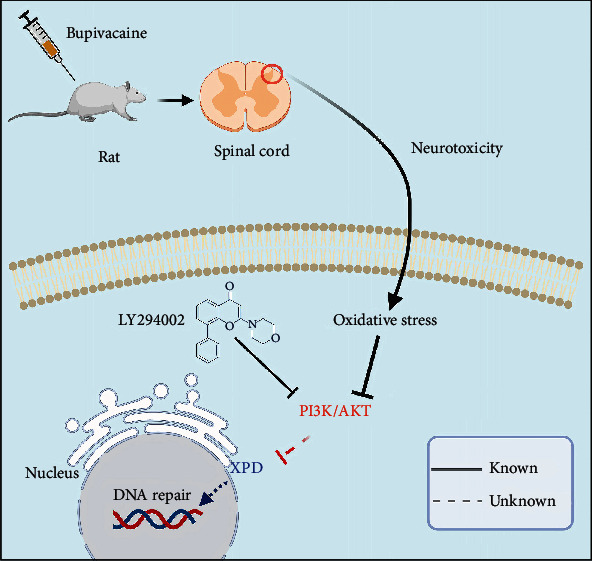
Graphical abstract. Bupivacaine may cause neuronal oxidative DNA damage. Damaged DNA activates a largely unknown repair mechanism. Our study showed that XPD is closely involved in repairing bupivacaine-induced oxidative DNA damage in neurons. Notably, XPD may be partially regulated by the PI3K/AKT pathway.

Ultimately, we drew a schematic of the proposed mechanism, which is shown in Figure 7: XPD, an enzyme of the NER pathway, may play an essential role in repairing bupivacaine-induced neuronal oxidative DNA damage. The repair enzyme XPD may be partially regulated by the PI3K/AKT signaling in response to DNA damage. These findings may be used as a reference for treating bupivacaine-induced neurotoxicity.

## Figures and Tables

**Table 1:** DNA repair gene expression in SH-SY5Y cells after exposure to bupivacaine was assessed with a cDNA plate array. The differentially expressed repair genes were mainly associated with the NER pathway.

C-*vs*-B (significant genes)	Pathways names	KEGG no.
SB, XPC-HR23B, XPD	Nucleotide excision repair	map03420
PTEN, RAD9, GADD45, ATR, P53	DDR(cell cycle)	map04110
DNA-PKcs	Nonhomologous end-joining	map03450
NTH1	Base excision repair	map03410

## Data Availability

All the data supporting the results were shown in the paper and can be available from the corresponding authors.

## Supplementary Materials

Supplementary Materials Supplemental Figure 1: validation of the lentivirus infection efficiency.
