# Supplementary material for: The DNA Repair Enzyme XPD Is Partially Regulated by PI3K/AKT Signaling in the Context of Bupivacaine-Mediated Neuronal DNA Damage
Source: Oxid Med Cell Longev. 2021 Oct 7;2021:9925647. doi: 10.1155/2021/9925647 (PMC8516563; doi:10.1155/2021/9925647)
Supplement: Supplementary Materials — Supplemental Figure 1: validation of the lentivirus infection efficiency. [file 9925647.f1.zip › Supplemental Figure 1.docx]

**
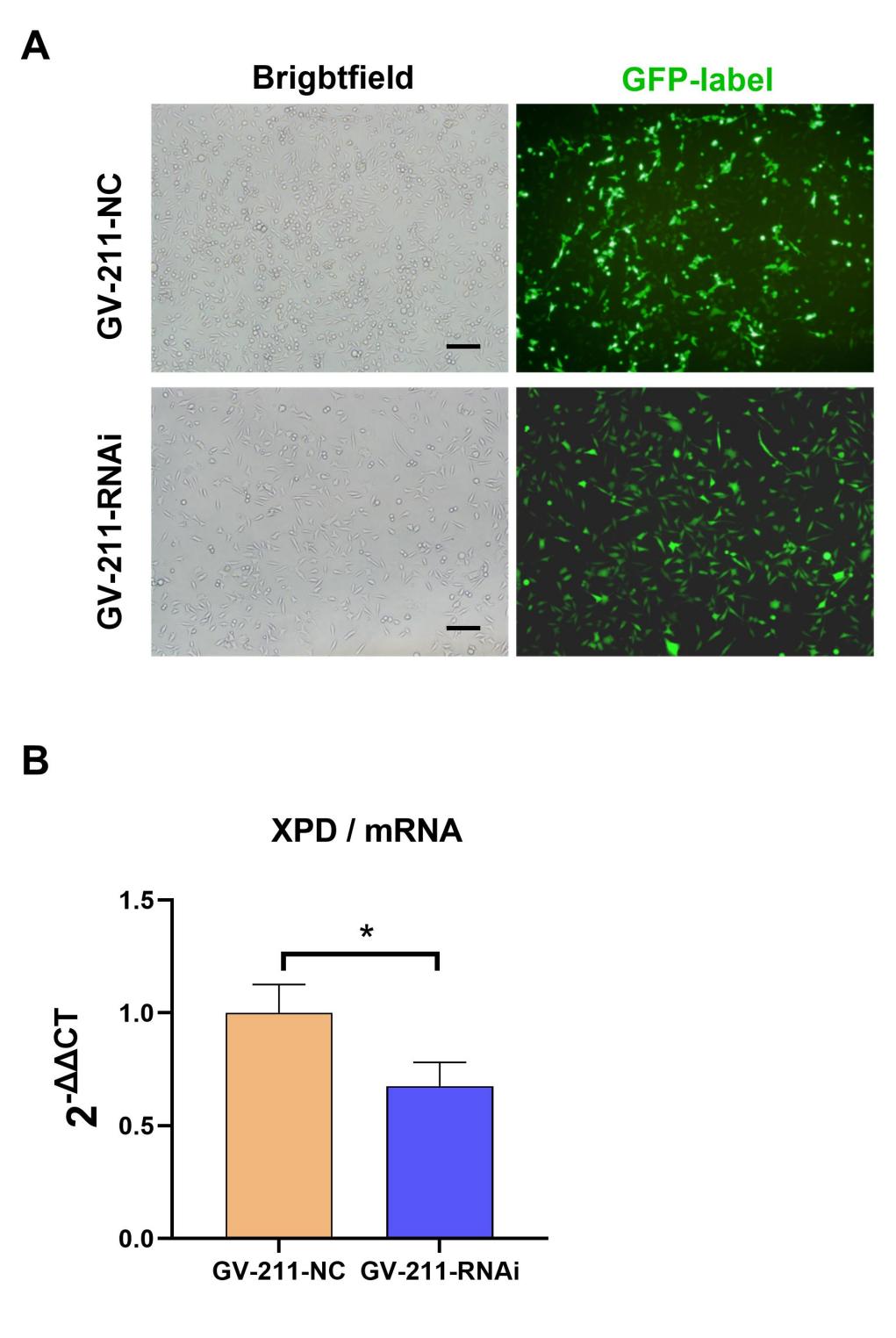
**

**Supplemental Figure 1. Validation of the lentivirus infection efficiency**

The fluorescence intensity was measured 48 h after SH-SY5Y cells were transfected with lentivirus. These images were used to determine the efficiency of lentivirus infection (A). Furthermore, Q-PCR was used to measure the mRNA levels of XPD in SH-SY5Y cells after lentivirus infection. Compared with that in the GV-211-NC-treated group, the mRNA expression of XPD in the GV-211-RNAi-treated group was significantly decreased (B) (n=3; **p*=0.0261).
