# Supplementary figures and images for: The DNA Repair Enzyme XPD Is Partially Regulated by PI3K/AKT Signaling in the Context of Bupivacaine-Mediated Neuronal DNA Damage
Source: Oxid Med Cell Longev. 2021 Oct 7;2021:9925647. doi: 10.1155/2021/9925647 (PMC8516563; doi:10.1155/2021/9925647)

**A**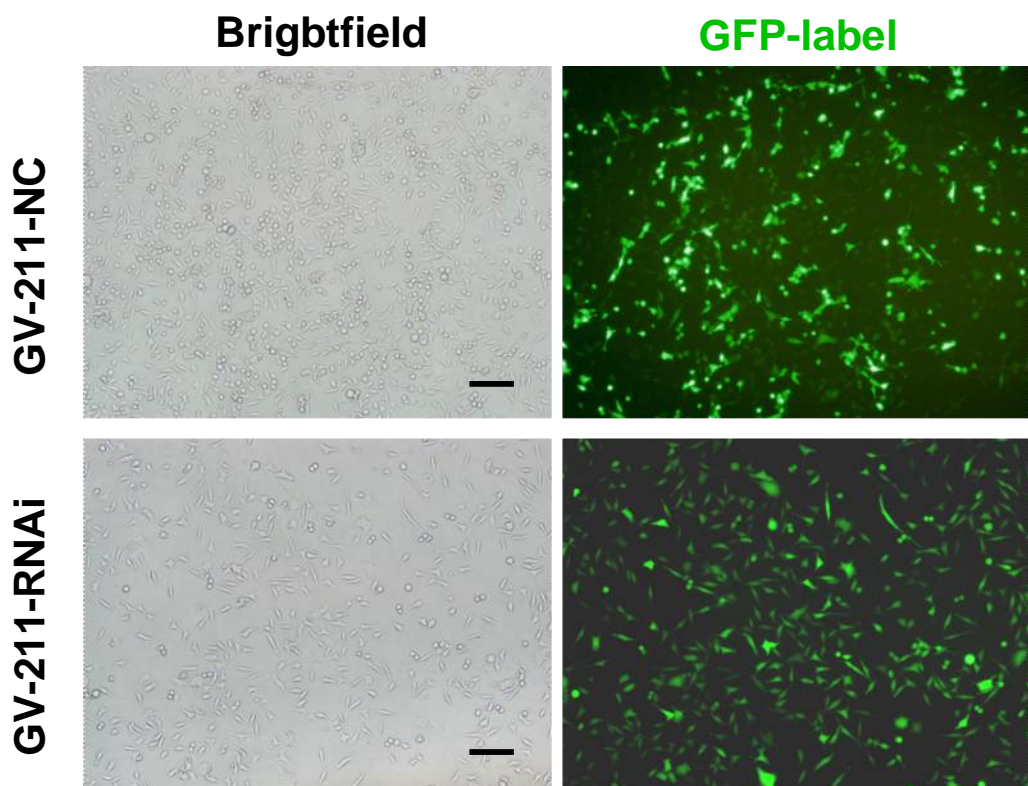**B**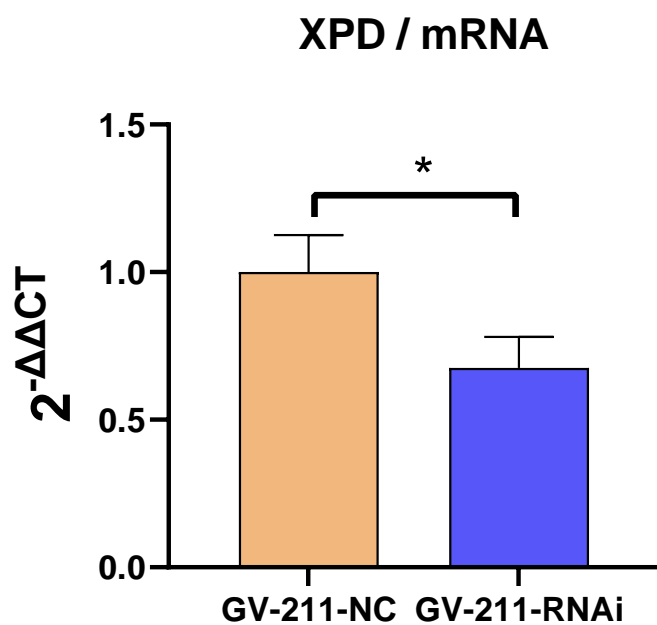

Supplement: Supplementary Materials — Supplemental Figure 1: validation of the lentivirus infection efficiency. [file 9925647.f1.zip › Supplemental Figure 1.pdf]
